# A plant-biotechnology approach for producing highly potent anti-HIV antibodies for antiretroviral therapy consideration

**DOI:** 10.1186/s43141-021-00279-z

**Published:** 2021-12-08

**Authors:** Advaita Acarya Singh, Priyen Pillay, Lusisizwe Kwezi, Tsepo Lebiletsa Tsekoa

**Affiliations:** Council for Scientific and Industrial Research, Future Production: Chemicals Cluster, P.O. Box 395, Pretoria, 0001 South Africa

## Abstract

Despite a reduction in global HIV prevalence the development of a pipeline of new therapeutics or pre-exposure prophylaxis to control the HIV/AIDS epidemic are of high priority. Antibody-based therapies offer several advantages and have been shown to prevent HIV-infection. Plant-based production is efficient for several biologics, including antibodies. We provide a short review on the work by Singh et al., 2020 who demonstrated the transient production of potent CAP256-VRC26 broadly neutralizing antibodies. These antibodies have engineered posttranslational modifications, namely *N*-glycosylation in the fragment crystallizable region and *O*-sulfation of tyrosine residues in the complementary-determining region H3 loop. The glycoengineered *Nicotiana benthamiana* mutant (ΔXTFT) was used, with glycosylating structures lacking β1,2-xylose and/or α1,3-fucose residues, which is critical for enhanced effector activity. The CAP256-VRC26 antibody lineage targets the first and second variable region of the HIV-1 gp120 envelope glycoprotein. The high potency of this lineage is mediated by a protruding *O*-sulfated tyrosine in the CDR H3 loop. *Nicotiana benthamiana* lacks human tyrosyl protein sulfotransferase 1, the enzyme responsible for tyrosine *O*-sulfation. The transient coexpression of the CAP256-VRC26 antibodies with tyrosyl protein sulfotransferase 1 in planta had restored the efficacy of these antibodies through the incorporation of the *O*-sulfation modification. This approach demonstrates the strategic incorporation of posttranslational modifications in production systems, which may have not been previously considered. These plant-produced CAP256-VRC26 antibodies have therapeutic as well as topical and systemic pre-exposure prophylaxis potential in enabling the empowerment of young girls and women given that gender inequalities remain a major driver of the epidemic.

## Introduction

Despite the global reduction in HIV prevalence, of the 38 million people living with human immunodeficiency virus/ acquired immunodeficiency syndrome (HIV/AIDS), only 25.4 million people are currently on antiretroviral treatment [1]. A compounding factor is gender inequality which remains a major social driver of the epidemic, with young women and adolescent girls accounting for one in four new infection in 2019 [1]. Thus, development of protective vaccines and a pipeline of new therapeutics or prophylaxes to control the HIV/AIDS epidemic remains a high priority [2, 3]. As an alternative or as a complement to small-molecule therapy such as highly active antiretroviral therapy (HAART) which utilizes small-molecule therapeutics (Table 1) in varying combination, antibody-based therapies have several advantages, such as safety and specificity [4]. The use of VRC01, a broadly neutralizing antibody (bNAb) has been shown to prevent HIV-infection in over 70% of people exposed to strains which are able to be neutralized by VRC01 [5].

**Table 1:** Food and Drug Administration (FDA)-approved small molecule HIV therapeutics

Drug type	Drug name
Nucleoside/nucleotide reverse transcriptase inhibitors	Abacavir, emtricitabine, lamivudine; tenofovir disoproxil fumarate, zidovudine
NNRTIs non-nucleoside reverse transcriptase inhibitors	Efavirenz, etravirine, nevirapine, rilpivirine
Fusion inhibitors	Enfuvirtide
Protease inhibitors	Atazanavir, darunavir, fosamprenavir, ritonavir, saquinavir, tipranavir
C-C chemokine receptor type 5 (CCR5)	Maraviroc
Integrase inhibitors	Dolutegavir, raltegravir, elvitegravir, bictegravir

The production of protein-based biopharmaceuticals has been dominated by the use of mammalian cell culture-based approaches [6]. There are alternative systems to mammalian-based production for protein-based biopharmaceuticals production; however, the ability of these non-mammalian systems to produce monoclonal antibodies (mAbs) is limited by their inherent properties. These properties comprise of cellular machinery which influence the ability to correctly fold both monomeric and multimeric structures and incorporate the correct post-translational modifications (PTMs) at the correct amino acids [7]. In contrast to other developing regions, the local manufacturing of protein-based vaccines and biopharmaceuticals is limited or growing at a slow rate in Africa thereby contributing to the widening trade deficit and limited access to essential medicines by the underprivileged [8]. Cost of goods, which will influence mass roll-out of such products, is also a major consideration [9]. Plant-based production is efficient for a number of biologics, and is particularly suitable for cost-sensitive markets in Africa and other low- and middle-income countries (LMICs) (reviewed by [10, 11]). The *Nicotiana benthamiana* (*N. benthamiana*) plant-based system has been employed for the production of anti-HIV antibodies, such as 2G12, VRC01, and PG9 [12–15], and the efficacy of some plant-produced versions have already been tested in animal trials [16].

We provide a short review on the paper entitled “Plant-based production of highly potent anti-HIV antibodies with engineered posttranslational modifications”. This publication reported the production of potent CAP256-VRC26 bNAbs with engineered PTMs in the antigen and fragment crystallizable (Fc) (receptor binding) region of the antibodies, respectively [17]. The ability to perform crucial *N*-glycosylation lacking β1,2-xylose and/or α1,3-fucose residues using glycoengineered *N. benthamiana* (ΔXTFT) plants complemented with the coexpression of the antibodies with human tyrosyl protein sulfotransferase 1 (hTPST1) had been demonstrated, thus enabling the *O*-sulfation of tyrosine residues in the complementary-determining region (CDR) H3 loop. The latter PTM is critical to the neutralization potency of the CAP256-VRC26 lineage of bNAbs.

## Glycoengineering of CAP256-VRC26 bNAbs

Glycosylation of the Fc region of Abs can significantly impact antibody effector functions like antibody-dependent cell-mediated cytotoxicity (ADCC) and antibody-dependent, cell-mediated virus inactivation (ADCVI) [18–20]. Wild type *N. benthamiana* glycosylates proteins with glycan species which are very different compared to mammalian production systems; proteins are produced with β1,2-xylose and/or α1,3-fucose containing *N*-glycans residues in wild-type *N. benthamiana* [21]. These *N*-glycan residues influence the pharmacokinetics of a biopharmaceutical product and effector functions. mAbs produced in *Lemna minor,* engineered to produce mAbs which lack β1,2-xylose and/or α1,3-fucose containing *N*-glycans residues, had demonstrated enhanced effector activity when compared with their Chinese hamster ovary (CHO)-derived homologs [22]. It is highly desirable to produce mAbs in plants which lack these β1,2-xylose and/or α1,3-fucose containing *N*-glycans residues [23, 24]. CAP256-VRC26.08 and CAP256-VRC26.09 were produced using a double knockout *N. benthamiana* mutant (ΔXTFT) which has attenuated expression of xylosyl- and fucosyltransferase via downregulation by ribonucleic interference (RNAi) resulting in the transient production of mAbs with a predominantly mammalian GnGn glycan structure [23, 24].

Incomplete glycosylation of the produced CAP256 bNAbs was observed [17], and this has been previously reported for other transiently plant-produced Abs [14, 15, 23, 25]. Higher glycosylation levels were observed in plant-produced VRC01 [14], which had led to assumption that these innate plant oligosaccharyltransferase (OST) complexes may not recognize the Fc glycosylation sites in different Abs with equivalent efficiency, resulting in the observed variations in glycosylation [13]. Increased in planta *N*-glycosylation can be achieved through the coexpression of recombinant protein with foreign OST subunits [13]. Glycosylation of the light chains of the CAP256 bNAbs produced in both mammalian and plant cells was observed [17]. The glycosylation of the light chains of Abs is known to shorten the clearance time of the antibody from blood [16]. These glycosylation sites can be removed to allow for increased blood circulation.

## *In planta* tyrosine *O*-sulfation of CAP256-VRC26 bNAbs

The CAP256-VRC26 antibody lineage targets the first and second variable region (V1V2) of the HIV-1 gp120 envelope glycoprotein. The high potency of this lineage alongside other V1V2 targeting mAbs, such as PG9, is mediated by a protruding *O*-sulfated tyrosine in the CDR H3 loop of the antigen-binding domain, a characteristic posttranslational modification (PTM) of V1V2 targeting antibodies [26–28]. The *O*-sulfated tyrosine of the CDR H3 loop facilitates tight binding of the gp120 envelope glycoprotein, in a manner which mimics the HIV-1 gp120 affinity for the sulfated chemokine receptor 5 (CCR5) [27]. Association of HIV-1 with the cluster of differentiation 4 (CD4) receptor and critically the CCR5 coreceptor, which has a sulfated tyrosine at the N-terminal end, is essential for HIV-1 gp120 binding and ultimately cell entry [27]. The absence of this modification in V1V2 targeting antibodies leads to a significant decrease in antigen-binding and results in loss of function [16]. The *O*-sulfation of tyrosine residues is carried out by hTPST1, an enzyme which *N. benthamiana* lacks. It was previously demonstrated that the transient coexpression of a V1V2 targeting antibody with hTPST1 in plants restored the efficacy of antibody through the proper incorporation of the *O*-sulfation modification [15]. The same transient coexpression strategy was used to incorporate the *O*-sulfation PTM to two tyrosine residues, Tyr112 and Tyr113, of which Tyr112 is critical to the efficacy of the CAP256-VRC26 bNAbs. Lower levels of sulfation were achieved in the plant-produced CAP256-VRC26 bNAbs with transient coexpression of hTPST1 when compared to the mammalian produced counterparts; however, despite the difference in sulfation, it was demonstrated that the plant-produced CAP256-VRC26 bNAbs with transient coexpression of hTPST1 have equivalent potency to that of their mammalian-produced counterparts. A similar level of sulfation was observed with PG9, which is indicative of transiently coexpressed hTPST1’s inability to efficiently sulfate tyrosines in the CDR H3 domain as the native machinery of the human embryonic kidney 293 (HEK293) cells [15]. *O*-sulfation levels may be improved through the further in vitro incubation of the CAP256-VRC26 bNAbs with hTPST1 and substrate, 3′-phosphoadenosine 5′-phosphosulfate (PAPS). However, this approach may not be viable at large scale. In any case, despite the difference in sulfation, equivalent efficacy between the plant-produced bNAbs with hTPST1 coexpression and mammalian-produced CAP256-VRC26 bNAbs counterparts.

## Proteolytic bottleneck of plant production of Abs

It was also noted that the plant-produced CAP256-VRC26 bNAbs were prone to proteolytic degradation [17]. A challenge faced with the production of proteins in *Nicotiana* species, is the proteolytic degradation of these recombinantly produced proteins in planta [29, 30]. The peptidase database, MEROPS, (27/07/2021) lists 515 known or putative peptidases and 98 non-peptidase homologs in *N. benthamiana* which may be responsible for in planta degradation of some recombinantly produced protein [31]. Proteolytic degradation may not only reduce the purity and yields of recombinantly produced protein but also compromises the structural integrity of these proteins. Proteolytic degradation such as this can result in altered biological activity or no protein production at all, ultimately resulting in a bottleneck in the production of biopharmaceuticals [32–34]. The antibodies are targeted through the plant secretory pathway for PTMs making them prone to proteolytic degradation by proteases which are auto-catalytically matured in low-pH environments [35, 36]. Plant-produced CAP256-VRC26 bNAbs were structurally similar to that of the mammalian produced bNAbs, despite the observation of proteolytic degradation fragments in the plant-produced CAP256-VRC26 bNAbs samples under reducing conditions.

The identity of the cleavage site/s are unknown, however, it was noted that under non-reducing conditions, no proteolytic degradation band is observed. Importantly, despite the presence of protease degradation products in the plant-produced Abs, similar neutralization potency was observed for the sulfated plant-produced Abs when compared to the mammalian-produced Abs. This suggests that this cleavage site does not influence antigen-binding in vitro. However, in vivo effects of such degradation are still unknown and will be a topic of our further research. The plant-produced CAP256-VRC26 bNAbs were structurally similar to that of the mammalian produced bNAbs. However, it may be important to improve the quality and/or quantity of produced mAbs in future. To circumvent such proteolytic degradation, commonly used strategies involve the use of RNAi to downregulate protease genes, or either the coexpression of plant protease inhibitors or proton channels to inhibit their enzymatic activity [37–40].

## Conclusion

Despite the success of current antiretroviral therapy, long term usage could introduce multiple drug-resistant escape mutants as a result of the high mutation rate and recombinant frequency of HIV [41]. Continuous development of HIV therapeutics which are both more effective and less toxic is essential [4]. The approach taken in our work [17] and others allows for the incorporation of strategic PTMs in production systems, which may have not been previously considered for the cost-effective production HIV antibody-based biotherapeutics. Gender inequalities remain a major driver of the epidemic with half of all new HIV infections in sub-Saharan Africa are among young people, with girls being two to three times more likely to be infected than boys [1]. Apart from the therapeutic potential of these plant-produced bNAbs, these bNAbs also have the potential to be used in topical and systemic pre-exposure prophylaxis (PrEP). It is thus prudent that work such as this be used in approaches which can enable the empowerment of young girls and women. The plant production system is likely to become increasingly important in enabling the production of antibodies and other proteins with therapeutic potential for mass roll-out in cost-sensitive markets where unequal access to resources, income opportunities, and social power drive high levels of HIV prevalence. The use of the plant-production system requires less capital expense/ investment and > 50% reduction in cost of goods than bioreactor-based processes, making it ideally suitable for LMICs [9]. This work has increased the likelihood that such plant-produced immunotherapeutic production strategies can be considered for adoption to prevent and treat HIV-1 infection in these markets, including sub-Saharan Africa.

## Data Availability

Not applicable.

## Abbreviations

HIV/AIDS: Human immunodeficiency virus/acquired immunodeficiency syndrome
HAART: Highly active antiretroviral therapy
bNAb: Broadly neutralizing antibody
LMICs: Low- and middle-income countries
*N. benthamiana*: *Nicotiana benthamiana*
Fc: Fragment crystallizable
ΔXTFT: Glycoengineered *N. benthamiana*
hTPST1: Human tyrosyl protein sulfotransferase 1
CDR H3: Complementary-determining region H3
PTM: Posttranslational modification
ADCC: Antibody-dependent cell-mediated cytotoxicity
ADCVI: Antibody-dependent, cell-mediated virus inactivation
CHO: Chinese hamster ovary
RNAi: Ribonucleic interference
OST: Oligosaccharyltransferase
V1V2: First and second variable region
CCR5: Chemokine receptor 5
CD4: Cluster of differentiation 4
Tyr: Tyrosine
HEK293: Human embryonic kidney 293
PAPS: 3′-phosphoadenosine 5′-phosphosulfate
